# A diet-specific microbiota drives *Salmonella* Typhimurium to adapt its in vivo response to plant-derived substrates

**DOI:** 10.1186/s42523-021-00082-8

**Published:** 2021-03-17

**Authors:** Nicoletta Prax, Stefanie Wagner, Jakob Schardt, Klaus Neuhaus, Thomas Clavel, Thilo M. Fuchs

**Affiliations:** 1 Lehrstuhl für Mikrobielle Ökologie, TUM School of Life Sciences, Technische Universität München, Weihenstephaner Berg 3, 85354 Freising, Germany; 2 ZIEL – Institute for Food & Health, Technische Universität München, Weihenstephaner Berg 1, 85354 Freising, Germany; 3 Friedrich-Loeffler-Institut, Institut für Molekulare Pathogenese, Naumburger Str. 96a, 07743 Jena, Germany; 4 Core Facility Microbiome, ZIEL – Institute für Food & Health, Technische Universität München, Weihenstephaner Berg 3, 85354 Freising, Germany; 5 Arbeitsgruppe Funktionelle Mikrobiomforschung, Institut für Medizinische Mikrobiologie, Uniklinik der RWTH Aachen, Pauwelsstrasse 30, 52074 Aachen, Germany

**Keywords:** Transcriptome, Gut microbiota, *Salmonella* Typhimurium, Diet, Metabolism

## Abstract

**Background:**

Little is known about the complex interactions between the diet, the gut microbiota, and enteropathogens. Here, the impact of two specific diets on the composition of the mouse gut microbiota and on the transcriptional response of *Salmonella* Typhimurium (*S.* Typhimurium) was analyzed in an enteritis model.

**Results:**

Mice were fed for two weeks a fibre-rich, plant-based diet (PD), or a Westernized diet (WD) rich in animal fat and proteins and in simple sugars, and then infected with an invasin-negative *S*. Typhimurium strain ST4/74 following streptomycin-treatment. Seventy-two hours post infection, fecal pathogen loads were equal in both diet groups, suggesting that neither of the diets had negatively influenced the ability of this ST4/74 strain to colonize and proliferate in the gut at this time point. To define its diet-dependent gene expression pattern, *S.* Typhimurium was immunomagnetically isolated from the gut content, and its transcriptome was analyzed. A total of 66 genes were more strongly expressed in mice fed the plant-based diet. The majority of these genes was involved in metabolic functions degrading substrates of fruits and plants. Four of them are part of the *gat* gene cluster responsible for the uptake and metabolism of galactitol and D-tagatose. In line with this finding, 16S rRNA gene amplicon analysis revealed higher relative abundance of bacterial families able to degrade fiber and nutritive carbohydrates in PD-fed mice in comparison with those nourished with a WD. Competitive mice infection experiments performed with strain ST4/74 and ST4/74 ΔSTM3254 lacking tagatose-1,6-biphosphate aldolase, which is essential for galactitol and tagatose utilization, did not reveal a growth advantage of strain ST4/74 in the gastrointestinal tract of mice fed plant-based diet as compared to the deletion mutant.

**Conclusion:**

A Westernized diet and a plant-based diet evoke distinct transcriptional responses of *S.* Typhimurium during infection that allows the pathogen to adapt its metabolic activities to the diet-derived nutrients. This study therefore provides new insights into the dynamic interplay between nutrient availability, indigenous gut microbiota, and proliferation of *S.* Typhimurium.

**Supplementary Information:**

The online version contains supplementary material available at 10.1186/s42523-021-00082-8.

## Background

*Salmonella enterica subsp. enterica* (*S. enterica*) is one of the most important model organisms to investigate bacterial genetics and pathogenicity. Its serovar Typhimurium (*S.* Typhimurium) is a Gram-negative, facultative anaerobic microorganism that causes non-thypoidal gastroenteritis in humans and typhoid-like disease in mice [1]. It is a food-borne pathogen that invades its host by contaminated food or water, eventually leading to salmonellosis [2]. During infection, *S.* Typhimurium needs sufficient energy as well as carbon and nitrogen sources to proliferate, colonize the epithelial barrier, produce virulence factors and withstand the host immune responses. A broad metabolic capacity is therefore a prerequisite for salmonellae to successfully compete with and outgrow commensal microorganisms in the terminal ileum and the colon. However, the acquisition of nutrients is a major challenge for *S.* Typhimurium due to colonization resistance of the commensal microbiota. This complex bacterial community forms a highly competitive environment via for instance the production of antimicrobial peptides and growth-inhibiting metabolites, and by reducing the amount of substrates freely available in the gut [3]. Moreover, as growth on only one carbon source is probably the exception for most bacteria invading the gut, *S.* Typhimurium is urged to rapidly switch from one nutrient to another, and thus to adapt its metabolic profile according to the metabolic status of each microenvironment encountered during infection.

The intestine of mammals and humans is a nutrient-rich reactor that is continuously fueled with substrates from the diet or, indirectly, with metabolites derived from the food or released from the mucus by bacterial enzymes [4]. Some of the main substrates in the gut are poly- and monosaccharides, fibres, proteins, starch, pectin, triglycerides, lactose, raffinose, creatine, mannan, xylan and cellulose. The indigenous microbiota produces approximately 9000 glycoside-hydrolases and 200 polysaccharide lyases [5], thereby aiding the host to optimally exploit dietary nutrients. This broad enzymatic capacity of commensal microbes results in the delivery of numerous simple sugars (glucose, xylose, galactose, fructose, mannose, arabinose, ribose), and many other metabolites such as phospholipids, glycerol, ethanolamine, lactate succinate, cholines, phenols, inositols, polyamines, indoles, short-chain fatty acids and other metabolites. Enzymes particularly produced by members of the phylum Bacteroidetes are able to digest mucus-derived glycans, thus providing galactose, gluconate, arabinose, fucose, rhamnose, 1,2-propanediol, sialic acid, N-acetyl-glucosamine, xylose, mannose, branched-chain fatty acids, di- and oligopeptides, and amino acids [4, 6]. Although such a wide range of nutrients is available in the gut, it is assumed that most metabolic niches, however, are already occupied by commensal microbes [7]. To overcome this metabolic competition, the gut-invading pathogen *S*. Typhimurium acquired and evolved specific metabolic capacities that include the utilization of sialic acid, fucose, melibiose, rhamnose, glycerol, ethanolamine, propanediol or *myo*-inositol [4, 8–12]. In the case of ethanolamine and 1,2-propanediol, both intestinal inflammation and the respiration of a microbiota-derived fermentation product enable this metabolic activity [13].

Any kind of perturbations that trigger substantial alteration of the gut microbiota can influence the conditions underlying bacterial growth and infection. Beside diseases and inflammation, antibiotic treatment and dietary changes are known to significantly interfere with the metabolic balance between host, gut microbiota and pathogens. Antibiosis usually strongly reduces microbial diversity and may thereby supports the expansion of non-targeted members of the gut microbiota. Moreover, colonization resistance is weakened by antibiotic treatment, resulting in an increased susceptibility to infection [14]. Nutritional changes have also been shown to change the structure and functions of gut microbial populations [15–18].

The complex and multiple interdependencies between the gut microbiota and an invading enteropathogen under varying dietary conditions remain to be elucidated in more detail. In particular, we tested the role of the gut microbiota as a key player in providing varying luminal conditions driven by different feeding protocols. For this purpose, we analyzed gut microbiota shifts and the resulting in vivo transcription patterns of *Salmonella* during proliferation in the gut of mice. We provide evidence that the transcriptional response of *S.* Typhimurium is diet-adapted, and that the diet-specific microbiota contributes to this adaptation by providing corresponding substrates.

## Results

### Effects of two experimental diets on the fecal microbiota structure

**Fig. 1:**
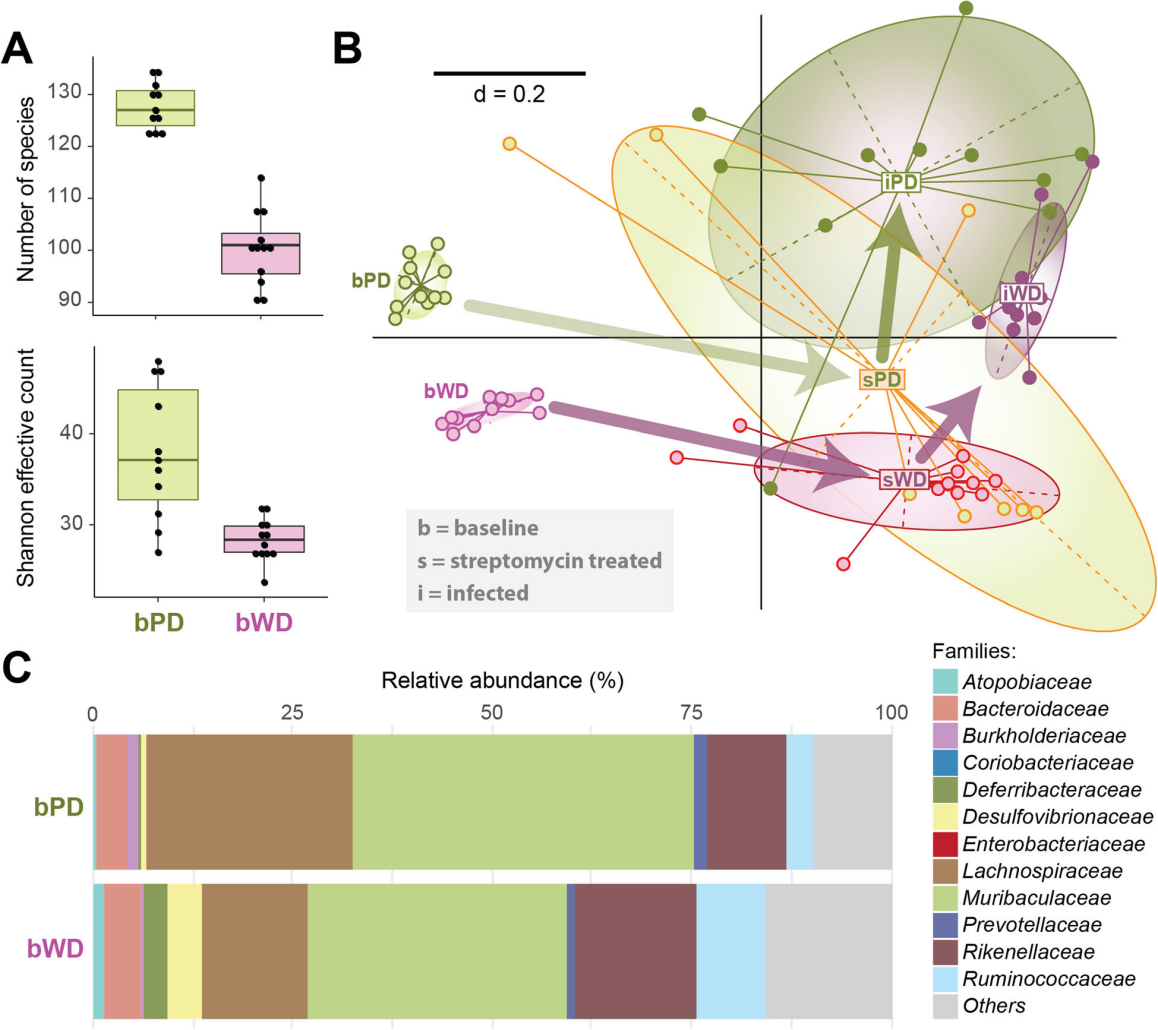
Differences in the fecal microbiota diversity and composition in to dietary mice groups. **a** Changes in α-diversity are shown as boxplots. All differences between two samples are significant (p ≤ 0.05) according to the pairwise Wilcoxon Rank Sum Test (n of WD fed mice = 12, n of PD fed mice = 11). **b** β-diversity analysis displayed as a nonmetric multidimensional scaling plot (metaNMDS) computed from generalized UniFrac distances, which were calculated from normalized OTU tables and phylogenetic distance trees (n of WD fed, untreated mice = 12, n of WD fed, streptomycin-treated/infected mice = 11, n of PD fed, untreated/infected mice = 11, n of PD fed, streptomycin-treated mice = 8). **c** An overview of relative abundances of major bacterial families is given by stacked bar plots. Cumulative abundances were calculated from all single OTUs classified within one family as per the best possible taxonomy using both the RDP and Silva. N of WD fed mice = 12, n of PD fed mice = 11

Twelve mice were fed either a Westernized diet (WD) or a plant-based diet (PD) for 13 days. Both diets had equal energy contents, and mice did not differ in terms of body weight at the time of feces collection (data not shown). The microbiota of PD fed mice were characterized by significantly higher species richness and higher Shannon effective counts compared with that of mice in the WD group (Fig. 1a). Non-metric multidimensional scatter (NMDS) plots of generalized UniFrac distances demonstrated clearly separated microbial phylogenetic makeup between the two dietary groups at baseline (Fig. 1b). In the fecal microbiota of untreated mice (baseline), eight different phyla were detected in both groups, of which *Bacteroidetes* (~ 61–64% relative abundance) and *Firmicutes* (~ 25–33%) were the most dominant. Family-level classification showed that members of the *Atopobiaceae* (phylum *Actinobacteria*) and *Deferribacteraceae* (represented by the species *Mucispirillum schaedleri*) were more abundant in WD mice (Fig. 1c**,** Additional files 1 and 2). The distribution of families within the phylum *Bacteroidetes* was also diet-dependent with a higher relative abundance of *Rikenellaceae* and *Tannerellaceae* in WD fed mice, whereas PD fed mice harbored predominantly the families *Prevotellaceae* and *Muribaculaceae*. The families *Lachnospiraceae* and *Burkholderiaceae* belonging to the phyla *Firmicutes* and *Proteobacteria*, respectively, exhibited higher proportions in the PD group, whereas *Ruminococcaceae* and *Desulfovibrionaceae* were more abundant in mice exposed to WD. The family *Anaeroplasmataceae* (phylum *Tenericutes*) occurred only in PD fed mice. Remarkably, the families *Prevotellaceae*, *Muribaculaceae*, and *Lachnospiraceae* are known to degrade carbohydrates such as fibers, hemicelluloses and starch, thus providing nutrients accessible for the host, other commensals and eventually invading pathogens.

These findings were confirmed and further differentiated by analyses at the level of OTUs (Additional files 2, 3, and 4). All differences in the microbiota composition described here and below were statistically evaluated as significant (*p* ≤ 0.05) according to the pairwise Wilcoxon Rank Sum Test and/or the Fisher’s Exact Test.

### In vitro phenotype of strain ST4/74 Δ*invA*

**Fig. 2:**
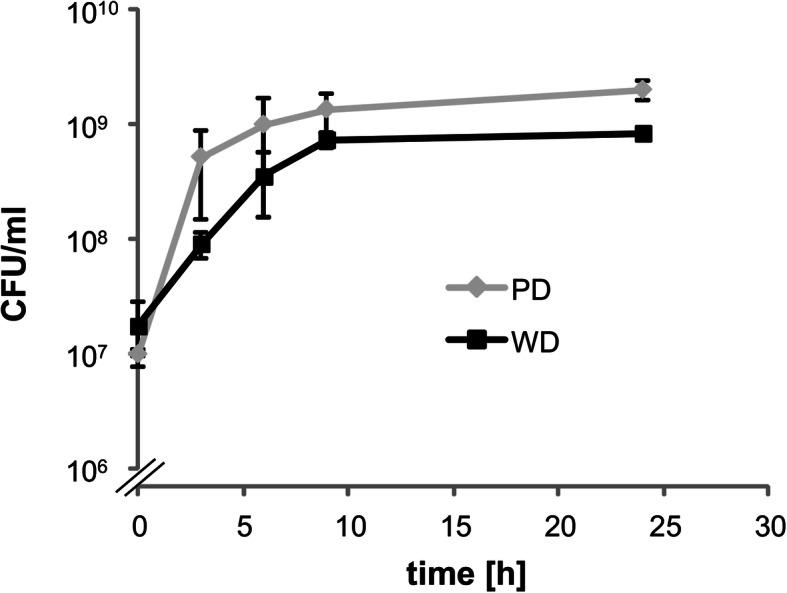
Growth phenotype of strain ST4/74 Δ*invA* in medium with PD and WD pellets. The growth behaviour of strain ST4/74 Δ*invA* was tested in medium with food pellets for PD (gray line) or WD (black line) dissolved in water. The CFU of three biological replicates were determined by plating three aliquots at each time point. Error bars show the standard deviation

To analyse the transcriptome of strain ST4/74 during infection, but independently of the hostʼs immune response, we constructed mutant ST4/74 Δ*invA* that is unable to invade epithelial cells, resulting in a retarded immune reaction [19–23]. To monitor effects of the two diets on growth of the mutant, diet pellets were mashed using sterile water and inoculated 1:100 with an overnight culture of ST4/74 Δ*invA* in LB medium. During incubation at 37 °C, aliquots of the cultures were taken and plated on LB agar plates to determine colony forming units (CFU). Starting with 1.0 ***×*** 10^7^ CFU/ml (PD) and 1.8 ***×*** 10^7^ CFU/ml (WD) immediately after inoculation, the cultures reached counts of 2.0 ***×*** 10^9^ CFU/ml (PD) and 8.4 ***×*** 10^8^ CFU/ml after 24 h. Slightly different growth properties were observed, indicating nearly equal growth of mutant ST4/74 Δ*invA* in the two diets (Fig. 2).

### Streptomycin treatment

**Fig. 3:**
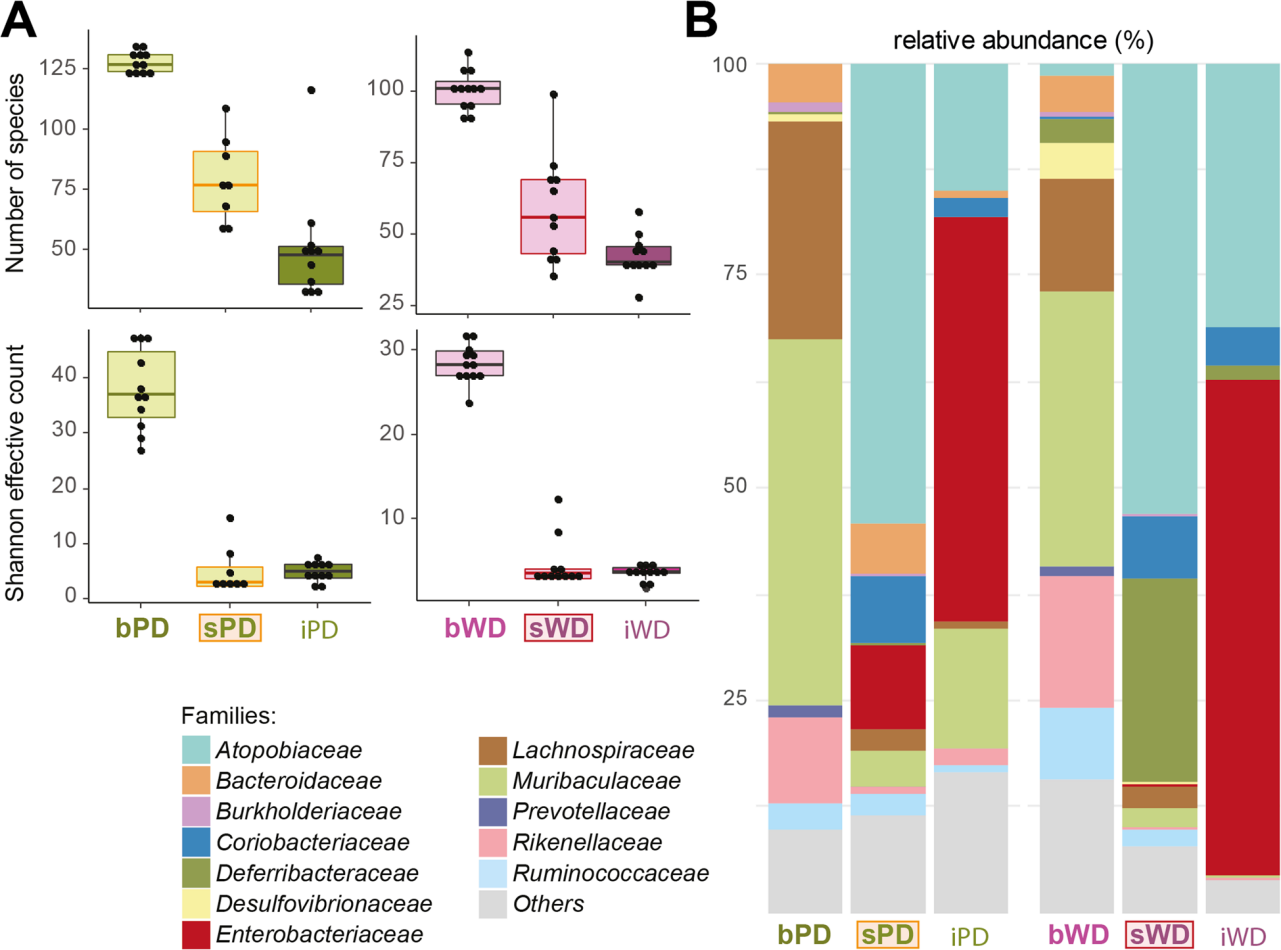
Microbial shifts in mice following streptomycin treatment and infection. **a** α-diversity changes are shown as boxplots. N of WD fed, untreated mice = 12, n of WD fed, streptomycin-treated/infected mice = 11, n of PD fed, untreated/infected mice = 11, n of PD fed, streptomycin-treated mice = 8. **b** For both PD or WD fed mice after streptomycin application and infection with *S.* Typhimurium, an overview of cumulative relative abundances of all dominant families are displayed. See legend of Fig. 1c for statistical details

Mice were treated with 20 mg streptomycin 24 h before infection to evoke a gastroenteritis and a retarded systemic infection, resulting in a higher susceptibility for and proliferation of *Salmonella* [24, 25]. Such a pathogen expansion is a prerequisite for an in vivo transcriptome analysis that requires a high cell number to isolate sufficient amounts of RNA. The treatment with streptomycin decreased species richness and Shannon effective counts (Fig. 3a) in feces independent of the diet, and the differences between the two diets remained equally significant. β-diversity analysis showed drastic shifts in the phylogenetic makeup of the microbiota after antibiosis (Fig. 1b). At the level of families, the application of streptomycin to both PD and WD fed mice markedly increased the relative abundance of *Atopobiaceae* and *Coriobacteriaceae*, whereas *Burkholderiaceae*, *Desulfovibrionaceae*, *Lachnospiraceae*, *Muribaculaceae*, *Prevotellaceae* and *Rikenellaceae* were sensitive to the antibiotic or/and were outcompeted by the other commensals. In the case of PD in particular, marked inter-individual differences were observed, indicating very diverse trajectories of changes following treatment. Here, but not in WD fed mice, streptomycin increased the proportion of *Anaeroplasmataceae* and *Bacteroidaceae*, and reduced that of *Erysipelotrichaceae*. In the intestine of WD fed mice, streptomycin treatment increased proportions of the family *Deferribacteraceae*, and decreased that of the families *Bacteroidaceae*, *Tannerellaceae* and *Ruminococcaceae* as compared with the PD fed group (Fig. 3b) (Additional files 1 and 5).

### Infection with ST4/74 Δ*invA*

**Fig. 4:**
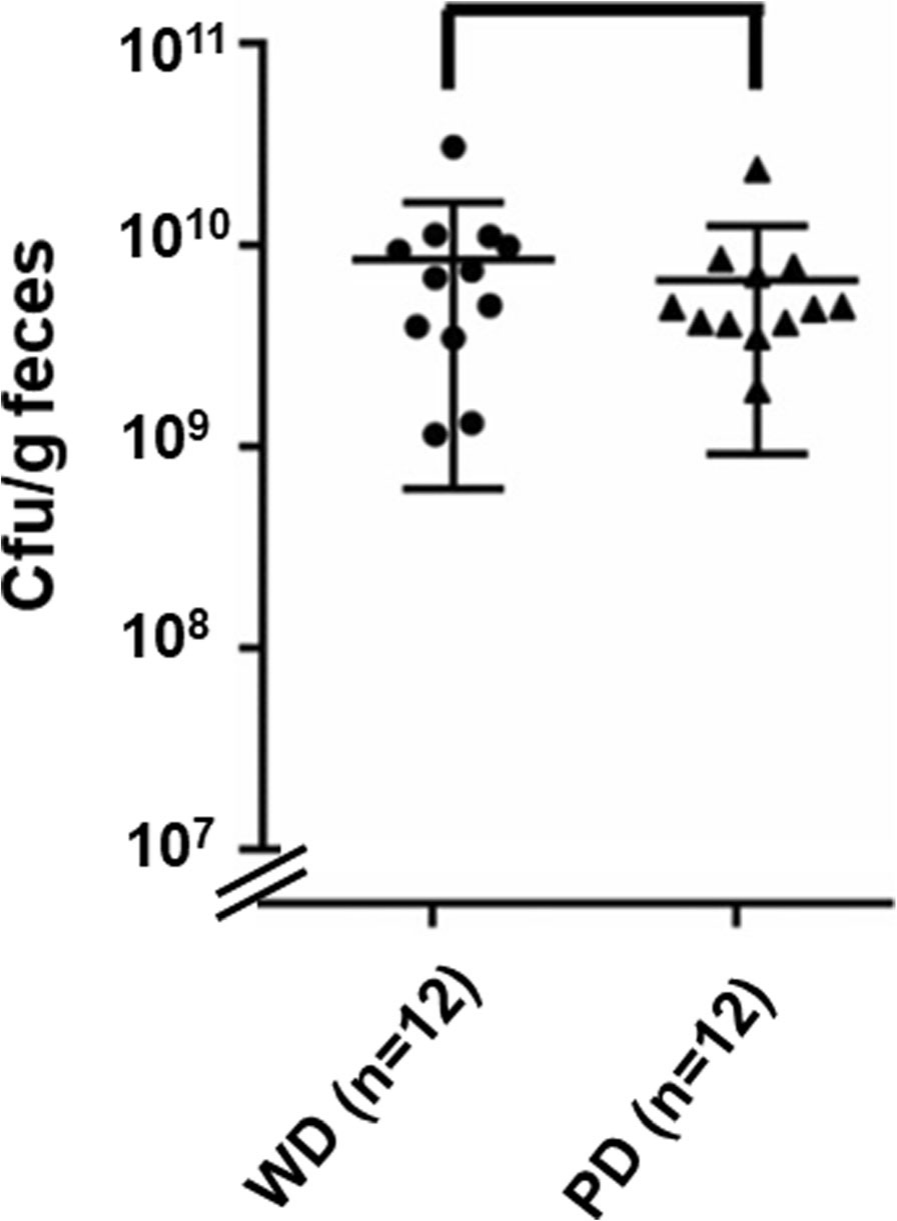
Diet-dependent load of *S.* Typhimurium 24 h post infection. Twelve mice per group were infected with 5 × 10^7^ cells of strain ST4/74 Δ*invA*. No significant difference between the averaged CFU were detected

Twenty-four h after the application of streptomycin, the mice were orally infected with 5 × 10^7^ ST4/74 Δ*invA* cells. After further 24 h, 10^9^–10^11^ *Salmonella* cells were found in the mouse gut, without significant differences in cell numbers between the two dietary groups (Fig. 4). The infection with *S.* Typhimurium caused a further decrease in species richness and shift of β-diversity of the fecal microbiota in both dietary groups in comparison with streptomycin-treated samples before infection (Fig. 1b**,** Fig. 3b). In line with the high number of *Salmonella* cells introduced into and proliferating in the gut, a high relative abundance of *Enterobacteriaceae*, the bacterial family to which *Salmonella* belongs, represented by 47.5% (PD) and 58.1% (WD) of all reads (Additional file 6), was observed 24 h after infection. Accordingly, the proportions of most other families decreased in both dietary groups, with the exception of *Coriobacteriaceae* in mice fed WD (Fig. 3b**,** Additional file 1).

### Validation of immunomagnetic separation to isolate *S*. Typhimurium from the gut content

As a prerequisite to analyze the in vivo transcriptome of *S.* Typhimurium ST4/74 Δ*invA*, we established a method to separate ST4/74 Δ*invA* cells from the commensal gut microbiota of mice by immunomagnetic separation (IMS) [26, 27]. We first tested *Salmonella*-specific antibodies (BacTrace; ViroStat, Maryland, ME, USA) for their selectivity and their cross-reactivity with related *Enterobacteriaceae*. *Salmonella* cells were separated from a 1:1 mixture of *Escherichia coli* DH5α/pBR322 and *S*. Typhimurium ST4/74 Δ*invA* by IMS, and the number of *S*. Typhimurium cells was determined before and after the separation by plating the suspensions on agar selective for *S*. Typhimurium ST4/74 Δ*invA* (Nal^R^) and *E. coli* DH5α/pBR322 (Tet^R^). A reduction of *E. coli* by 99.5% was achieved using the BacTrace antibody, whereas no significant separation of both bacteria was obtained using the ViroStat antibody. Even when the *Salmonella* and the *E. coli* strain were mixed at a ratio of 1:10.000, ST4/74 Δ*invA* cells were enriched to a final ratio of 11:1 following IMS with the BacTrace antibody, indicating its high binding specificity. Then, ST4/74 Δ*invA* cells were mixed with cecum content isolated from mice to concentrations of 1.57 × 10^6^ CFU/ml, 5.36 × 10^7^ CFU/ml and 3.78 × 10^8^ CFU/ml, with the highest concentration corresponding to that found in the caecum of mice 20 h after infection [28]. Applying IMS, approximately 21, 33 and 41%, respectively, of the *Salmonella* cells were retrieved. We also inoculated the caecum content with a 60:40 mixture of *E. coli* DH5α/pBR322 and *S*. Typhimurium ST4/4 and noted a reduction of *E. coli* to 10% after one IMS separation and to 3% of all bacterial cells after a second application of the antibodies. These data validated IMS for the separation of *Salmonella* cells from the gut of infected mice after infection.

### In vivo transcriptome of *S*. Typhimurium

Cecum and ileum content of mice infected with strain ST4/74 Δ*invA* were dissected together to isolate *Salmonella* cells via IMS 24 h after infection. The mice did not show signs of illness. The time point was chosen to reduce possible inflammation that might interfere with diet-dependent effects. After RNA isolation, a cDNA library was constructed and sequenced. We identified 66 *Salmonella* genes that were differentially regulated in mice fed with one of the two diets in comparison with the other one. Thirty-eight of these genes were found to be more strongly induced in ST4/74 Δ*invA* infected mice fed PD than in those fed WD. Of those 38 genes, the remarkably high number of 29 belong to the categories transport and metabolism of carbohydrates. This includes genes responsible for the metabolism of arabinose and the uptake and/or utilization of glycolate, fructose, sorbitol, tagatose, galactitol, rhamnose and melibiose (Table 1). All these sugars and polyols are found in plants, and the upregulated genes directly reflect the higher amount of plant material in the PD. In addition, genes involved in arginine metabolism (*arcA*, *arcC*, *argF*) and in sialic acid catabolism (*nanA*), and four genes attributed to energy production and conversion revealed to be upregulated in mice fed predominantly with plant material.

**Table 1:**
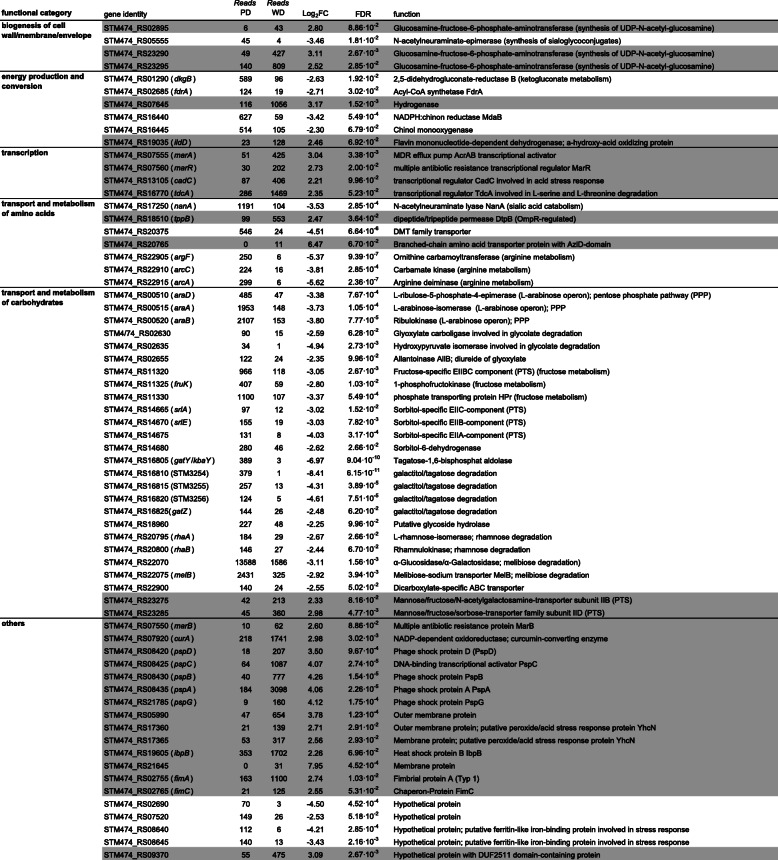
Transcriptome analysis of strain ST4/74 isolated from mice fed WD or PD. FDR, false discovery rate. Positive log_2_ fold-changes (FC) (gray) indicate a higher gene expression in WD fed mice in comparison with the PD fed group, negative FC (white) vice versa

In contrast, 28 ST4/74 genes were upregulated following infection of mice fed WD as compared to the other dietary group. Many of these genes are involved in stress response and encode phage shock proteins PspA-D and PspG, the heat shock protein IbpB, the acid stress response regulator CadC, and two putative proteins responding to peroxide or acid stress (Table 1). Remarkably, the multiple antibiotic resistance (*marRAB*) operon, three genes homologous to glucosamine-fructose-6-phosphate-aminotransferase genes, two genes involved in mannose/sorbose transport, and two genes involved in the production of fimbriae were also found to be upregulated under this condition. Taken together, a PD results in a specific induction of metabolic genes in *S.* Typhimurium, whereas a WD predominantly activates stress genes.

### The *gat* operon contributes to tagatose utilization

**Fig. 5:**
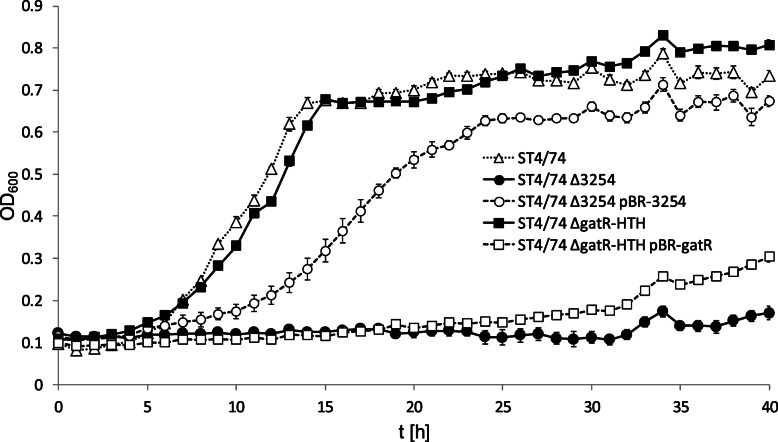
Tagatose-dependent growth phenotypes of *S*. Typhimurium. Growth curves of strains ST4/74, ST4/74 ΔSTM3254, ST4/74 ΔSTM3254/pBR-3254, ST4/74 Δ*gatR*-HTH, and ST4/74 Δ*gatR*-HTH/pBR-*gatR* in MM with 1% D-tagatose as sole carbon source. Standard deviations of three replicates are shown

Five of the *Salmonella* genes upregulated in mice fed PD are part of the *gat* operon known to be responsible for galactitol utilization [29]. Three of the gene products of the *gat* operon are predicted to be involved in tagatose metabolism (Table 1). We deleted one of them, gene STM3254 encoding a hypothetical tagatose-1-phosphate kinase, which is essential for galactitol utilization, and tested its growth in MM with tagatose as sole carbon and energy source. Under this condition, mutant ST4/74 ΔSTM3254 exhibited a zero growth phenotype that could be complemented by providing the gene *in trans* using plasmid pBR-3254 (Fig. 5). When we tested another mutant, ST4/74 Δ*gatR*-HTH (Table 2), which is not able to repress the *gat* promoters due to a lack of the nucleotides of *gatR* encoding the DNA-binding site of the repressor GatR [29], a growth behavior with tagatose similar to that of parental strain ST4/74 was observed. This finding is in line with the assumption that the *gat* promoters are activated in the presence of galactitol and tagatose. Upon complementation of ST4/74 Δ*gatR*-HTH with plasmid pBR-*gatR*, growth was inhibited probably due to the high number of repressor molecules. Taken together, we could demonstrate that the *gat* operon of *S.* Typhimurium is responsible for D-tagatose utilization, and that mutant ST4/74 ΔSTM3254 is unable to degrade this sugar.

**Table 2:** Strains and plasmids used in this study

Strains	Description and relevant features	references
*S.* Typhimurium ST4/74	Nal^R^	[30]
ST4/74 Δ*invA*	Non-polar *invA* deletion mutant	This study
ST4/74 ΔSTM3254	Non-polar STM3254 deletion mutant with zero growth phenotype in galactitol	[29]
ST4/74 ΔSTM3254::KanR	Mutant with allelic exchange of STM3254 against a kanamycin resistance gene	This study
ST4/74 Δ*gatR*-HTH	Partial non-polar deletion of *gatR* lacking the nucleotides encoding the helix-turn-helix motif (HTH)	This study
*E. coli*		
DH5α	F^–^ *endA1 glnV44 thi-1 recA1 relA1 gyrA96 deoR nupG purB20* φ80ΔlacZΔM15 Δ(lacZYA-argF )U169, hsdR17 (*r*_*K*_^–^*m*_*K*_^+^), λ^–^	
**Plasmids**		
pBR322	Amp^R^, Tet^R^	[31]
pBR-STM3254	pBR322 with gene STM3254	This study
pBR-*gatR*	pBR322 with repressor gene *gatR*	This study
pKD4	Kan^R^, *pir*-dependent, FRT sites	[32]
pKD46	λ-Red helper plasmid, Amp^R^	[32]
pCP20	FLP recombinase plasmid, Cm^R^, Amp^R^	[32]

### Galactitol utilization does not provide a growth advantage of *S.* Typhimurium in C57/BL6J

**Fig. 6:**
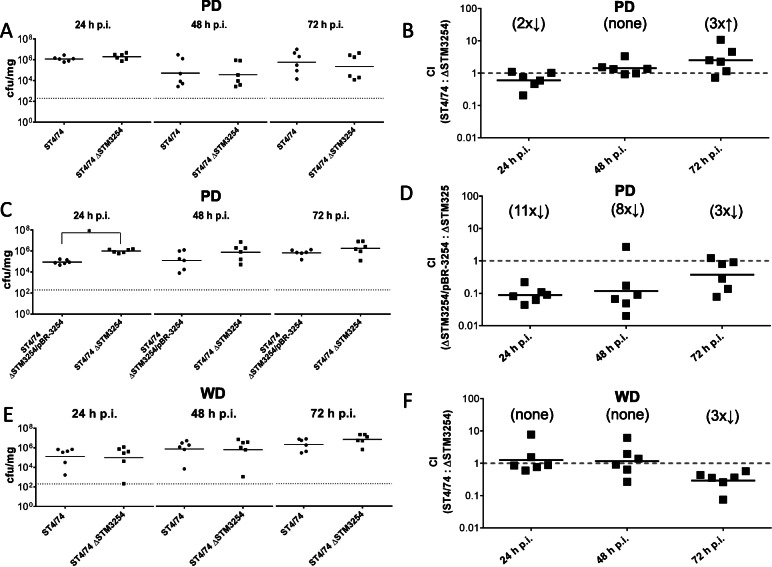
Deletion of STM3254 leads to reduced infection numbers in C57/BL6J mice over time. Two groups of female C57/BL6J mice (n = 6 each group) were fed the PD or the WD and orally infected with a 1:1 ratio of ST4/74 and ST4/74 ΔSTM3254::Kan^R^, and on group (*n* = 6) was fed the PD and infected with a 1:1 ration of ST4/74 ΔSTM3254::Kan^R^ and ST4/74 ΔSTM3254::Kan^R^ /pBR-3254. The total inoculum was 5 × 10^7^ CFU/mouse. Stool samples were taken every 24 h up to 3 days (**a**, **c**, **e**). The CFU per mg feces of each strain are shown over time. The numbers of ST4/74 (circles) ST4/74 ΔSTM3254::Kan^R^ (squares) (or ST4/74 ΔSTM3254::Kan^R^ (circles) and ST4/74 ΔSTM3254::Kan^R^ /pBR-3254 (squares) recovered in each mouse after co-infection are depicted. Solid horizontal lines indicate mean values while dashed lines lines represent the detection limit for each sample. **b**, **d**, **f**) CIs depict the ratio of ST4/74 and ST4/74 ΔSTM3254::Kan^R^, or ST4/74 ΔSTM3254::Kan^R^ and ST4/74 ΔSTM3254::Kan^R^ /pBR-3254). The geometric mean for each group was compared to the theoretical value of 1.0 and the fold-change difference is indicated in parentheses. Significant differences (*p* value ≤0.05) are indicated by asterisks

We hypothesized that the ability to metabolize galactitol and/or tagatose enhances the ability of *S.* Typhimurium strain ST4/74 to compete with gut microbiota and to persist in the intestinal lumen following oral transmission. To test this, female C57/BL6J mice were co-infected with a 1:1 ratio of ST4/74 and an isogenic mutant ST4/74 ΔSTM3254, or with a 1:1 ratio of ST4/74 ΔSTM3254 and ST4/74 ΔSTM3254/pBR-3254 (total of 5 × 10^7^ CFU/mouse) that were differentially tagged with antibiotic resistances. Recombinant pBR322 retains its stability in *S*. Typhimurium in vivo [33]. Two groups were fed the PD, and one group was fed WD (*n* = 6 per group). Feces were collected daily for up to 3 days p.i., and the total number of strain per mg of feces and the competitive index (CI) was determined. In stool from PD-fed mice, about 10^6^ CFU of *S.* Typhimurium were recovered after 24 h p.i. with the average number of ST4/74 ΔSTM3254 mutant bacteria being slightly higher than the wild type strain (Fig. 6a). At 48 h p.i., the CFU of both strains reached equal levels, and after 72 h numbers of ST4/74 surpassed the ST4/74 ΔSTM3254 mutant. This was also reflected by the calculated CI values, which increased over time (Fig. 6b). Surprisingly, the complementation strain ST4/74 ΔSTM3254/pBR-3254 was recovered in significantly (*p* < 0.0017) lesser numbers as compared to the ST4/74 ΔSTM3254 mutant at 24 h p.i. (Fig. 6c). This discrepancy in the CFU decreased from an 11-fold to a 3-fold difference over time as indicated by the increasing CI values (Fig. 6d). Looking at stool from WD-fed mice, the average CFU of ST4/74 ΔSTM3254 and the wild type strain were equal at 24 h and 48 h p.i., (Fig. 6e) as indicated by CI values of 1 (Fig. 6f). At 72 h p.i., the ST4/74 ΔSTM3254 mutant was recovered in 3-fold higher fecal numbers than ST4/74. The CFU for both strains steadily increased over time, in contrast to mice fed PD. Taken together, the absence of the genes encoding the galactitol utilization pathway did not cause a significant phenotype in the mouse gastrointestinal tract under plant-derived feed, although the *gat* operon is specifically upregulated in mice fed this diet. This finding suggests that other metabolic pathways compensate for the metabolic deficiency of the mutant strain.

## Discussion

Diet modifications, antibiotic therapy or chronic gut diseases can lead to a substantial change in microbial gut communities. This might enable non-native enteric pathogens to overcome barriers created by the communities of commensals and thus successfully persist and colonize the gut. Distinct differences in dietary formula have often been associated with alterations in the composition of gut microbial populations [34], particularly regarding WD versus Mediterranean, vegan or PD [35–39]. Here, we investigated the effect of two diets containing either monosaccharides as well as protein and fatty acids of animal origins (WD), or polysaccharides and plant-based proteins and fatty acids (PD), on the transcriptional response of *S.* Typhimurium, taking into account changes affecting the gut microbiota of mice.

Fecal microbiota analysis of feces revealed substantial differences of the two dietary groups at the level of major taxonomic groups. PD fed mice were characterized by high relative abundances of the family *Muribaculaceae* (43.01%), many members of which can degrade a variety of complex carbohydrates [40], and also of *Prevotellaceae* spp. able to hydrolyse hemicellulose. Another major group of commensal bacteria involved in fiber degradation, the *Lachnospiraceae*, was also more abundant in PD fed mice (25.61%) than in those exposed to WD (13.10%). Although *Firmicutes* are often associated with a high intake of animal-associated foods [36], their role in polysaccharide breakdown has been underestimated [41], an assumption that might explain their prevalence in mice fed PD. Vice versa, a higher proportion of *Rikenellaceae* which was observed to thrive on high-fat diets [17], was detected in the microbiota of mice fed WD. Taken together, the gut microbiota profiles were dependent on the composition of the two diets. We particularly hypothesize that the microbiota of PD fed mice provided plant-derived substrates that are accessible for *S.* Typhimurium and feed its metabolism during infection. Subsequent treatment with streptomycin lowered the colonization resistance due to partial depletion of commensal microbes, particularly of facultative anaerobes [14, 42], and allowed an expansion of *S.* Typhimurium for the benefit of the in vivo transcriptome. Although this experimental step possibly perturbed the digestion of the diet, the post-streptomycin analysis of the microbiota demonstrated that families involved in the degradation of carbohydrates were still present in the mouse gut (Additional file 5).

The main question in our study was whether or not the enteropathogen *S.* Typhimurium responds specifically to distinct diets. We separated the *S*. Typhimurium cells 24 h after infection from the commensal bacteria using immunomagnetic beads and analyzed their transcriptome. To exclude that the IMS procedure, which took approximately 30 min, impacts the results, the gut content was transferred into RNA later, and all IMS steps were performed at 4 °C if possible.

We hypothesized that at this early stage of infection by an invasion-negative strain, at least a part of the transcriptional response is associated with metabolic properties reflecting the pathogen’s efforts to adapt to the substrate availability in the mouse gut. Strikingly, the majority of the *Salmonella* genes upregulated in mice fed PD belongs to the categories transport and metabolism of carbohydrates and amino acids. According to their upregulation, plant-derived substrates like glycolate, fructose, sorbitol, tagatose, galactitol, rhamnose, and melibiose are used by the enteropathogen to fulfill its metabolic needs. Thus, the upregulation of the respective utilization pathways in mice fed PD, but not in rodents fed WD, points to a metabolic adaptation of *S*. Typhimurium to the plant-rich diet and the substrates derived thereof by the digestive activity of the host and its commensal microbiota. For example, melibiose is a disaccharide composed of glucose and galactose and, together with fructose, a cleavage product of another plant sugar, raffinose.

The utilization of mucosal carbohydrates by bacterial pathogens, especially after antibiotic treatment, has been described for *Clostridium difficile*, *S. enterica*, *Listeria monocytogenes*, and *E. coli* [13, 43–46]. Although we identified *nanA* involved in sialic acid catabolism, the large gene cluster responsible for the degradation of fucose [4] was missing in the list of differentially regulated *Salmonella* genes in our study. Possible reasons are the short infection time of one day and a depletion of commensal bacteria such as *Bacteroides thetaiotaomicron*, which are specialized to cleave fucose from glycans of the gut mucus.

While *S.* Typhimurium ST4/74 specifically responded to PD by the upregulation of genes involved in carbohydrate utilization, no specific metabolic adaptation was observed to the high concentration of fat in WD fed mice. This suggests that sucrose (33.44% in the WD and 5% in the PD) is a readily usable energy source for *S*. Typhimurium that is preferred over metabolizable proteins or fat. In contrast to most of the above-mentioned plant-derived carbohydrates, the relevance of tagatose and galactitol for in vivo replication of *S*. Typhimurium had not been investigated so far. Galactitol is a reduction product of galactose, a common sugar in the gut lumen. Tagatose is obtainable by microbial oxidation of galactitol and a degradation product of galactosamine and N-acetylgalactosamine, both present in the intestinal mucin. Therefore, both substrates are considered to be part of the hostʼs galactose metabolism [47–49]. The *gat* operon was demonstrated by gene deletions to link galactitol degradation [29] with tagatose utilization (this study). Interestingly, transposon-directed knockout of three genes of the *gat* operon resulted in an attenuated colonization of chicken, calves and pigs by *S*. Typhimurium strain ST4/74, supporting the role of galactitol and tagatose utilization in vivo [50]. Furthermore, tagatose plays an anti-diabetic role by controlling the blood glucose level. Therefore, the upregulation of the genes responsible for tagatose utilization particularly in mice fed PD points to a microbiota composition in the animals of the respective dietary group that may help to decrease the risk of diabetes [49].

The total CFU of *S.* Typhimurium in the two dietary groups seventy-two hours p.i. did not differ significantly from each other, but an approximately 10-fold increase of CFU from 24 h to 72 h was observed in the group fed WD. An even 10^2^–10^5^ higher stool pathogen load reported recently for mice reared with a high-fat WD in comparison to the control group [51]. The competitive infection experiments performed in our study using mutants with a deletion of gene STM3254 and its complementation did not support the relevance of galactitol utilization for *S*. Typhimurium in vivo, although the *gat* operon was specifically upregulated in mice fed the PD. However, we had chosen to use a setting without streptomycin treatment, in which the microbiota is not reduced and confers colonization resistance. It can therefore not be excluded that galactitol degradation by *S.* Typhimurium provides a significant growth and colonization advantage in other infection models, or that other carbohydrates are utilized by *S*. Typhimurium to compensate for this deficiency.

The colitis model based on streptomycin treatment [25] was applied here to overcome colonization resistance conferred by the C57/BL6J microbiota to obtain high bacteria numbers for the RNA sequencing. Given that this treatment significantly alters the microbial composition as demonstrated here, we cannot exclude that the microbiota dysbiosis at least partially affected the diet-dependent transcriptional response of *S*. Typhimurium assessed here. It is also known that gastrointestinal colonization evokes a significant gastrointestinal inflammation starting eight hours post infection [28], resulting in gut oxygenation. To avoid or reduce inflammation that might distort the transcriptional response of *S.* Typhimurium to the diets, we used an *invA* deletion mutant with reduced capacity to invade epithelial cells, thus not resulting in tissue inflammation [20]. Although gut inflammation cannot be excluded to play a role in our experimental setting, we hypothesize here that the specific transcriptome of strain ST4/74 is indeed mainly triggered by the distinct metabolic properties of a diet-driven microbiota, which, however, exhibited a reduced species richness due to streptomycin treatment.

## Conclusion

During infection, salmonellae encounter the colonization resistance of the gut that is, among others, based on the limitation of nutrients due to metabolic niche occupation by the commensal microbiota. *S. enterica* have acquired specific metabolic adaptations that help them to overcome this hurdle. However, the influence of the diet and the gut microbiota on the metabolic behaviour of this pathogen is largely unknown. This study therefore investigated the interaction between diet, gut microbiota composition, and the transcriptional response of *Salmonella enterica* during mice infection. To our best knowledge, this is the first study that describes the in vivo transcriptome of salmonellae separated from the gut microbiota. We show that a plant-based diet, in concert with the microbiota composition, specifically provokes the activation of metabolic pathways of *Salmonella* involved in the utilization of substrates that are derived from fruits and plants. This confirms the assumption that *S*. Typhimurium possesses a robust metabolism able to adapt to diverse metabolic niches and conditions, and that substrates beside galactitol are used by the pathogen in the gut lumen [52]. To conclude, the findings described here go a step forward in deciphering the metabolic adaptation of an enteropathogen to the specific nutrient conditions shaped by the complex interaction between diet and microbiota.

## Methods

### Growth of bacterial strains

The strains and plasmids used in this study are listed in Table 2. *S.* Typhimurium strain 4/74 (ST4/74) was grown in lysogenic broth (LB: 10 g/L tryptone, 5 g/L yeast extract, 5 g/L NaCl) or in minimal medium (MM) consisting of M9 medium supplemented with 2 mM MgSO_4_, 0.1 mM CaCl_2_ and 55.5 mM (1% w/v) tagatose. If appropriate, the media were supplemented with the following antibiotics: kanamycin, ampicillin (50 μg/ml each), nalidixic acid (20 µg/ml), or tetracycline (12 µg/ml). For solid media, 1.5% agar (w/v) was added. For all growth experiments, bacterial strains were grown in LB medium overnight at 37 °C and inoculated 1:100 in the desired liquid growth medium. Growth curves were obtained from bacterial cultures incubated at 37 °C without agitation in 15 ml falcon tubes with 10 ml medium. Colony-forming units (CFU) per ml were counted by plate streaking.

### Standard molecular techniques

DNA manipulations and isolation of chromosomal and plasmid DNA were performed according to standard protocols [53], and following the manufacturersʼ instructions. GeneRuler™ DNA Ladder Mix (Fermentas, St. Leon-Rot, Germany) was used as a marker for DNA analysis. Plasmid DNA was transformed via electroporation using a Bio-Rad Gene pulser II as recommended by the manufacturer and as described previously [54]. Polymerase chain reactions (PCRs) were carried out with Taq polymerase (Fermentas). As template for PCR, chromosomal DNA, plasmid DNA, or an aliquot of a single colony resuspended in 100 μl H_2_O was used. Oligonucleotides used in this study are listed in Additional file 7. Genes *invA*, STM3254 and *gatR*-HTH were deleted using the λ-Red recombinase [32]. Briefly, PCR products containing the kanamycin resistance cassette of plasmid pKD4 and the flanking FRT sites were generated using primers of 70 nucleotides in length that included 20 nucleotides priming sequences for pKD4 as template DNA. The fragments were transformed into ST4/74 cells harboring plasmid pKD46, and the allelic replacement of the target genes was controlled by PCR. A nonpolar deletion mutant was obtained by transformation with pCP20 and validated by PCR analysis and DNA sequencing.

### Mouse infection assays

Female C57/BL6J mice at the age of 6–10 weeks obtained from in-house breeding at the Kleintierforschungszentrum Weihenstephan (Freising, Germany) were transferred and maintained in a specific-pathogen-free facility with a 14-h light and 10-h dark cycle. After one week of acclimatization, mice (*n* = 12 per group) were fed either a Westernized diet or a plant-based diet (both from ssniff, Soest, Germany) (Table 3). After two weeks, mice were treated with 20 mg streptomycin by gavage 24 h before the infection. Mice were orally infected by gavage with 5–8 × 10^7^ ST4/74 Δ*invA*.

**Table 3:** Diet composition

		TD88137 – Westernized diet (WD)	S5745-E750 – plant-based diet (PD)
Casein	%	19.5	–
Cholesterol	%	0.21	–
Isolate of soy protein	%	–	5
Concentrate of soy protein	%	–	20
Sucrose	%	33.44	5
Maize starch	%	–	41.79
Cellulose	%	5	7
Lignocellulose	%	–	3
Fructooligosaccharide /Chicorée inulin	%	–	2
Appel marc	%	–	4
DL-Methionine	%	0.3	0.2
Mix of mineral and trace elements	%	4.3	3.5
Mix of vitamines	%	1	1
Calcium	%	0.76	–
Calciumcarbonate	%	–	0.3
Cholin Cl	%	0.2	0.2
Ascorbic acid	%	0.1	–
Butylhydroxytoluol	%	0.01	0.01
Butter fat	%	21	–
Soybean oil	%	–	7
Approximate composition of nutrients			
Protein	%	17.5	17.5
Fat	%	21.2	7.3
Fibres	%	5	12.7
Mineral elements	%	4.5	4.9
Starch	%	14.6	40.8
Sugar	%	33.2	7.2
Calcium	%	0.76	0.73
Phosphor	%	0.46	0.51
Sodium	%	0.37	0.25
Magnesium	%	0.1	–
Potassium	%	0.54	–
Fatty acids			
C 4:0	%	0.8	–
C 6:0	%	0.53	–
C 8:0	%	0.29	–
C 10:0	%	0.63	–
C 12:0	%	0.72	–
C 14:0	%	2.21	–
C 16:0	%	5.74	0.84
C 17:0	%	0.13	0.01
C 18:0	%	2.04	0.25
C 20:0	%	0.04	0.03
C 16:1	%	0.38	0.01
C 18:1	%	4.63	1.8
C 18:2	%	0.38	3.8
C 18:3	%	0.11	0.42
C 20:1	%	0.02	–
Metabolizable energy	MJ/kg	19.2	14.6
Protein	kJ%	15	20
Fat	kJ%	42	19
Carbohydrates	kJ%	43	61

To perform competitive infections, overnight cultures of ST4/74, ST4/74 ΔSTM3254::Kan^R^ and ST4/74 ΔSTM3254::Kan^R^/pBR-3254 were adjusted to 5 × 10^8^ CFU/ml, and 1:1 mixtures of two strains (ST4/74 and ST4/74 ΔSTM3254::Kan^R^, or ST4/74 ΔSTM3254::Kan^R^ and ST4/74 ΔSTM3254::Kan^R^/pBR-3254) with a total of 5 × 10^7^ CFU/mouse were used for oral infection of female C57/BL6J mice by gavage. Two group of mice were fed the PD, and one group the WD (*n* = 6 per group). Sample collection and handling was performed as follows: One to three stool pellets were collected every 24 h, weighed and suspended in sterile PBS. Samples were homogenized in a FastPrep-24 benchtop homogenizer (MP Biomedicals, Eschwege, Germany) using 1 mm (Ø) silica beads (Sigma-Aldrich, St. Louis, MO, USA). Serial dilutions were plated on *Salmonella*-*Shigella*-agar (Roth, Karlsruhe, Germany) containing the appropriate antibiotics and incubated for 24–48 h.

### Isolation of *S.* Typhimurium cells from the gut of infected mice

Animals were sacrified 24 h after infection. Cecum and ileum content were transferred into Eppendorf tubes containing 1 ml RNAlater (Thermo Fisher Scientific, Langenselbold, Germany) and 0.1 mm (Ø) Zirkonia/Silica beads (BioSpec Products, Bartlesville, OK, USA) prior to storage at − 80 °C. After thawing, 100 μl TritonX-100 were added, and the gut preparations were homogenized with a ribolyser (MP Biomedicals, Eschwege, Germany). The gut content was passed through three filters with a pore size of 100 μm, 70 μm and 30 μm (Miltenyi Biotech GmbH, Bergisch Gladbach, Germany), respectively, and the bacteria were pelleted by centrifugation at 6500×g for 5 min. The pellet was resuspended in 1 ml buffer (1 × PBS, 0.5% biotin-free BSA) containing 10% (v/v) RNAlater. Ten μl of *Salmonella*-specific antibodies (BacTrace, KPL Inc., Maryland, USA) diluted 1:10 were added, and the mixture was incubated with shaking at 4 °C for 10 min. The sediment, obtained by centrifugation at 9600×g for 2 min, was washed with cold separation buffer (1 × PBS, 0.5% biotin-free BSA, 2 mM EDTA pH 7.4) containing 10% RNAlater, centrifuged, resuspended in the same buffer and incubated with 10 μl of streptavidin-coupled magnetic beads for 15 min. The washing and centrifugation steps from above were repeated, and the sediment was resuspended in 500 μl separation buffer containing 10% RNAlater. *S*. Typhimurium cells were separated using a MACS cell separation system with a LS coloumn (Miltenyi Biotech, Aubum, CA, USA) according to the manufacturer’s instructions.

### RNA isolation

RNA was extracted and purified from 1 ml of a *Salmonella* suspension isolated from cecum using a NucleoSpin® RNA kit (Macherey-Nagel GmbH & Co. KG, Düren, Deutschland) according to the manufacturerʼs instruction. Briefly, the bacterial pellet was solved in 100 μl TE buffer with 1 mg/ml lysozyme and incubated for 10 min at 37 °C. For further cell lysis, 350 μl RA1 buffer and 3.5 μl β-mercaptoethanol were added and vortexed vigorously. The lysate was filtrated for 1 min at 11,000 x *g*, and 350 μl of 70% ethanol were added. The RNA was bound to a column via centrifugation for 30 s at 11,000 x *g*, and 350 μl MDB was added to desalt the silica membrane, followed by centrifugation for 1 min at 11,000 x *g*. 95 μl of a reaction mixture containing 10 μl rDNase and 90 μl reaction buffer were applied onto the silica membrane of the column and incubated at room temperature for 15 min. The membrane was successively washed with 200 μl buffer RAW2 (30 s at 11,000 *g*), and with 600 μl and 250 μl buffer RA3 (30 s at 11,000 x *g* and 2 min at 11,000 x *g*, respectively). Finally, the RNA was eluted in 60 μl of RNase-free H_2_O. RNA quality was assessed using a 2100 Bioanalyser (Agilent, Waldbronn, Germany).

### Transcriptome analysis

Whole-transcriptome RNA library preparation with isolated RNA was performed as described [55]. Briefly, ribosomal RNAs were depleted using the RiboMinus Transcriptome Isolation Kit (Invitrogen, Darmstadt, Germany), and RNA was fragmented via sonication using a Covaris sonicator. After dephosphorylation and rephosphorylation, the TruSeq Small RNA Sample Kit (Illumina, Munich, Germany) was used, and the resulting cDNAs were size-selected using polyamide-gel electrophoresis. Libraries were then diluted and sequenced on a MiSeq sequencer (Illumina, Munich, Germany) using a MiSeq Reagent Kit v2 (50 cycles), resulting in 50 bp single-end reads. Illumina FASTQ files were mapped to the reference genome of *S*. Typhimurium ST4/74 (GenBank: CP002487.1) using Bowtie2 for Illumina implemented in Galaxy [56, 57]. Artemis was used to visualize and calculate the number of reads mapping on each gene [58, 59]. Gene counts of each library were normalized to the smallest library in the comparison and reads per kilobase per million mapped reads (RPKM) values were calculated. Fold changes between the different conditions were calculated.

### Sequencing of 16S rRNA gene amplicons

Mice feces were collected in 600 μl DNA stabilization solution (STRATEC biomedical) and frozen at − 20 °C. After thawing on ice, 400 μl phenol:chloroform:isoamyl alcohol (25:24:1; Sigma-Aldrich) and about 500 mg 0.1 mm glass beads (Roth) were added. Microbial cells were lyzed using a FastPrep-24 (MP Biomedicals) fitted with a 24 × 2 ml cooling adaptor for 3 × 30 s at maximum speed. After a short heat treatment (95 °C, 5 min) and centrifugation (15,000×g, 5 min, 4 °C), supernatants were treated with RNase A (0.1 μg/μl, 30 min, 37 °C). Complete DNA was purified using gDNA columns (Macherey-Nagel) following the manufacturer’s instructions. DNA was controlled using a NanoDrop photometer and a Qubit fluorometer (Thermo Scientific). The V3-V4 region of 16S rRNA genes were amplified in a two-step PCR following [60], starting with 12 ng of metagenomic DNA. The primers 341F and 785R [61] fitted with overhangs were used in the first PCR for 15 cycles. The second PCR was conducted with barcoded primers for additional 15 cycles. Amplicons were purified using AMPure XP (Beckmann) and pooled. The pool was amended with 15% PhiX standard and sequenced (paired end, 2 × 300 cycles) using a MiSeq system (Illumina, Inc.) following the manufacturer’s instructions.

### Sequencing data analysis

To analyse the 16S rRNA gene sequencing data, raw reads were processed with the Integrated Microbial Next Generation Sequencing (IMNGS) pipeline [62] based on UPARSE [63]. Sequences were demultiplexed, trimmed to the first base with a quality score < 3, and then paired. Assemblies with a size < 250 and > 600 nucleotides or an expected error > 3 were excluded. Remaining reads were trimmed by ten nucleotides at each end to prevent the analysis of regions with distorted base composition. The presence of chimeras was tested with UCHIME [64]. Operational taxonomic units (OTUs) were clustered at 97% sequence identity, and only those with a relative abundance ≥0.5% in at least one sample were kept. Taxonomies were assigned at 80% confidence level by taking into account results from both the RDP classifier [65] and SILVA using SINA (v1.2.11) [66]. All further analyses were performed in the R programming environment using Rhea [67], following scripts and instructions available online (https://lagkouvardos.github.io/Rhea/). A PERMANOVA test (vegan::adonis) is performed in each case to determine if the separation of sample groups is significant, as a whole and in pairs. Counts are, by standard, normalized via simple division to their sample size and then multiplication by the size of the smaller sample, thus avoiding to introduce random variance or loss of data. The filtered and normalized OTU table used as basis for all analyses is provided in the Supplemental Material (Additional file 3). β-diversity was computed based on generalized UniFrac distances [68]. α-diversity was assessed on the basis of species richness and Shannon effective diversity [69] as explained in detail in Rhea. *P* values were corrected for multiple comparisons according to the Benjamini-Hochberg method. Only taxa with a prevalence ≥30% (proportion of samples positive for the given taxa) in one given group were considered for statistical testing.

To analyse the transcriptome data, the fastq data files were uploaded on the Galaxy server (https://usegalaxy.org/) to map the sequencves via Bowtie2 on the genome of *S.* Typhimurium ST4/74 (accession numbers CP002487-CP002490). Read sorting by coordinates was done via the picard software (http://sourceforge.net/projects/picard/files/picard-tools). Statistical analysis of the data was performed within R.

### Statistics and data evaluation

Statistical analyses for all experiments were performed using the Student’s *t*-Test with Welch’s correction, which is less influenced by unequal sample sizes, in Prism6 (GraphPad, La Jolla, CA, USA). P values ≤0.05 were considered as mentioned in the text.

## Supplementary Information

**Additional file 1 Fig. S1.** Overview of relative abundances of major bacterial genera shown as stacked bar plots. Cumulative abundances were calculated from all single OTUs classified within one genus as per the best possible taxonomy using both the RDP and Silva. N of WD fed, untreated mice = 12, n of WD fed, streptomycin-treated/infected mice = 11, n of PD fed, untreated/infected mice = 11, n of PD fed, streptomycin-treated mice = 8.

**Additional file 2 Table S1.** Significant influences of two diets on the microbiota composition.

**Additional file 3 Table S2.** OTU table.

**Additional file 4 Table S3.** OTU sequences.

**Additional file 5 Table S4.** Significant influences of streptomycin on the microbiota composition.

**Additional file 6 Table S5.** Significant influences of the *S.* Typhimurium infection on the microbiota composition.

**Additional file 7 Table S6.** Oligonucleotides used in this study.

## Data Availability

Raw sequence data are available at the European Nucleotide Archive (Microbiota data: SRA accession PRJNA560458, BioSample accessions SAMN12586589, SAMN12586590, SAMN12586591, SAMN12586592, SRR9983045; transcriptomic data: SRA accession: PRJNA560458, BioSample accessions SAMN12593626, SAMN12593627, SAMN12593628, SAMN12593629, SAMN12593630, SAMN12593631). All other data generated or analysed during the current study are included in the manuscript and its supplementary files.

## Abbreviations

PD: Plant-based diet
CFU: Colony forming units
WD: Westernized diet
NMDS: Non-metric multidimensional scatter
OTUs: Operational taxonomic units
