# Supplementary figures and images for: A diet-specific microbiota drives Salmonella Typhimurium to adapt its in vivo response to plant-derived substrates
Source: Anim Microbiome. 2021 Mar 17;3:24. doi: 10.1186/s42523-021-00082-8 (PMC7972205; doi:10.1186/s42523-021-00082-8)

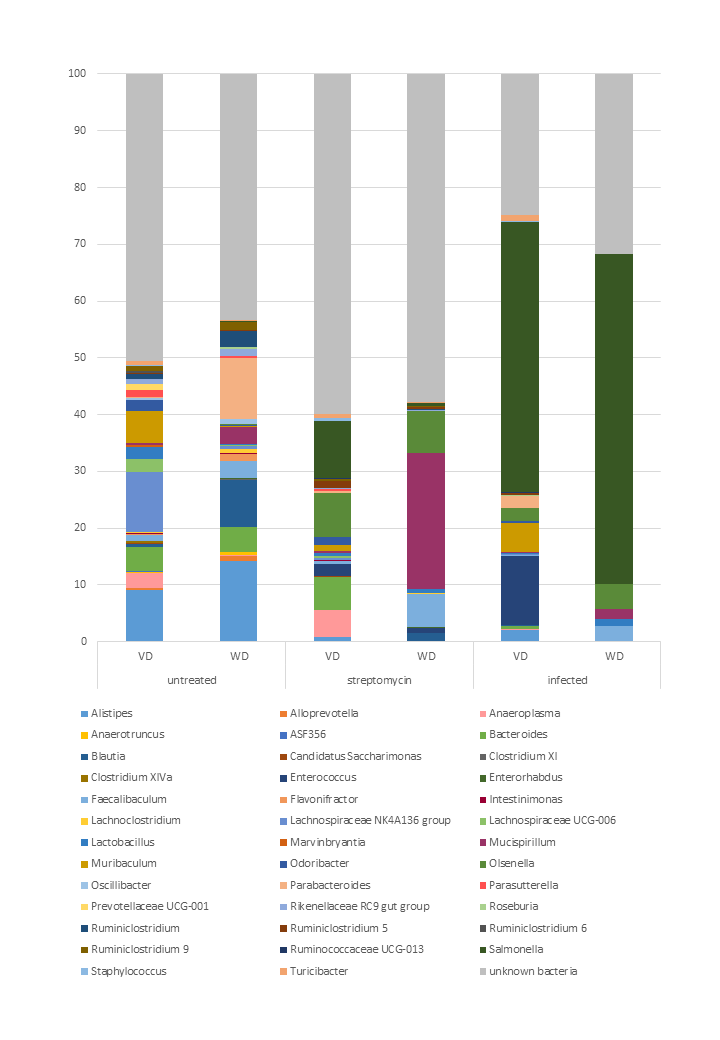

Supplement: Supplementary file 1 — Additional file 1 Fig. S1. Overview of relative abundances of major bacterial genera shown as stacked bar plots. Cumulative abundances were calculated from all single OTUs classified within one genus as per the best possible taxonomy using both the RDP and Silva. N of WD fed, untreated mice = 12, n of WD fed, streptomycin-treated/infected mice = 11, n of PD fed, untreated/infected mice = 11, n of PD fed, streptomycin-treated mice = 8. [file 42523_2021_82_MOESM1_ESM.tif]
