# Supplementary material for: A diet-specific microbiota drives Salmonella Typhimurium to adapt its in vivo response to plant-derived substrates
Source: Anim Microbiome. 2021 Mar 17;3:24. doi: 10.1186/s42523-021-00082-8 (PMC7972205; doi:10.1186/s42523-021-00082-8)
Supplement: Supplementary file 2 — Additional file 2 Table S1. Significant influences of two diets on the microbiota composition. [file 42523_2021_82_MOESM2_ESM.pdf]

TABLE S1. Significant influences of two diets on the microbiota composition. W, Wilcoxon rank sum test; F, Fisher's exact test; n, number of mice underlying statistic calculation; SD, standard deviation; WD, Westernized diet; PD, plant-based diet.

Phylum level

Phylum	*p* value (W)	*p* value (F)	WD			PD		
			mean [%]	n	SD [%]	mean [%]	n	SD [%]
Actinobacteria	0.0242		1.79	8	1.53	0.32	3	0.21
Cyanobacteria		0.0373	0.66	5	0.88		0	
Deferribacteres	0.0011	0.0013	2.98	12	1.43	0.35	4	0.28
Proteobacteria	0.0001		5.17	12	2.05	2.11	11	0.56
Tenericutes		0.0001		0		2.57	9	2.61

Class level

Phylum	Class	*p* value (W)	*p* value (F)	WD			PD		
				mean [%]	n	SD [%]	mean [%]	n	SD [%]
Actinobacteria	*Coriobacteriia*		0.0361	1.59	8	1.35	0.29	2	0.18
Cyanobacteria	*Melainabacteria*		0.0373	0.66	5	0.88		0	
Deferribacteres	*Deferribacteres*	0.0011	0.0013	2.98	12	1.43	0.35	4	0.28
Proteobacteria	*δ-Proteobacteria*	0.0013	0.0137	4.24	12	2.03	0.71	6	0.63
	γ-Proteobacteria		0.0137	0.46	6	0.30	1.24	11	0.63
Tenericutes	*Mollicutes*		0.0001		0		2.57	9	2.61

Family level

Phylum	Family	*p* value (W)	*p* value (F)	WD			PD		
				mean [%]	n	SD [%]	mean [%]	n	SD [%]
Actinobacteria	*Atopobiaceae*		0.0272	1.48	7	1.34	0.26	1	
Bacteroidetes	*Marinifilaceae*		0.000	0.14	1		1.95	11	0.63
	Muribaculaceae	0.0156		32.42	12	4.71	43.01	11	11.18
	Prevotellaceae	0.0056		1.00	9	0.52	1.62	9	0.69
	Rikenellaceae	0.0156		15.50	12	6.51	9.92	11	1.76
	Tannerellaceae		0.0000	10.63	12	8.47	0.26	1	
Cyanobacteria	*unknown. Gastranaerophilales*		0.0373	0.66	5	0.88		0	
Deferribacteres	*Deferribacteraceae*	0.0011	0.0013	2.98	12	1.43	0.35	4	0.28

Firmicutes	*Lachnospiraceae*	0.0156		13.10	12	5.98	25.61	11	12.51
	Ruminococcaceae	0.0000		8.49	12	3.18	3.28	11	1.61
Proteobacteria	*Burkholderiaceae*		0.0046	0.39	5	0.25	1.17	11	0.59
	Desulfovibrionaceae	0.0013	0.0137	4.24	12	2.03	0.71	6	0.63
Tenericutes	*Anaeroplasmataceae*		0.0001		0		2.57	9	2.61
**Genus level**									
Phylum	Genus	*p* value (W)	*p* value (F)	WD			PD		
				mean [%]	n	SD [%]	mean [%]	n	SD [%]
Bacteroidetes	*Alistipes*		0.0225	14.18	12	6.33	9.08	11	1.64
	Muribaculum		0.0000		0		5.66	11	1.38
	Odoribacter		0.0000	0.14	1		1.95	11	0.63
	Parabacteroides		0.0000	10.63	12	8.47	0.26	1	
	Prevotellaceae UCG−001		0.0000		0		1.17	9	0.57
Deferribacteres	*Mucispirillum*	0.0011	0.0013	2.98	12	1.43	0.35	4	0.28
Firmicutes	*Blautia*	0.0044	0.0003	8.29	12	4.33	0.45	3	0.43
	Faecalibaculum	0.0030		3.09	12	2.01	1.00	8	0.50
	Flavonifractor		0.0006	1.27	11	0.50	0.28	2	0.26
	Lachnospiraceae NK4A136 group		0.0000	0.47	1		10.59	11	4.86
	Lachnospiraceae UCG−006		0.0000		0		2.30	10	1.39
	Ruminiclostridium 9	0.0138		1.45	12	0.54	0.86	10	0.32
	unbek. *Ruminiclostridium*	0.0012		2.87	11	2.11	0.80	7	0.47
	Turicibacter		0.0123	0.17	2	0.18	0.84	8	0.58
Proteobacteria	*Parasutterella*		0.0046	0.39	5	0.25	1.17	11	0.59
Tenericutes	*Anaeroplasma*	0.0001			0		2.57	9	2.61
