# Supplementary material for: A diet-specific microbiota drives Salmonella Typhimurium to adapt its in vivo response to plant-derived substrates
Source: Anim Microbiome. 2021 Mar 17;3:24. doi: 10.1186/s42523-021-00082-8 (PMC7972205; doi:10.1186/s42523-021-00082-8)
Supplement: Supplementary file 5 — Additional file 5 Table S4. Significant influences of streptomycin on the microbiota composition. [file 42523_2021_82_MOESM5_ESM.pdf]

TABLE S4. Significant influences of streptomycin on the microbiota composition. W, Wilcoxon Rank Sum Test; F, Fisher's Exact Test; n, number of mice harboring species; SD, standard deviation; WD, Westernized diet; PD, plant-based diet.

**Family level (WD)**								
Family	*p* value (W)	*p* value (F)	untreated			streptomycin treated		
			mean [%]	n	SD [%]	mean [%]	n	SD [%]
Atopobiaceae	0.01	0.09	1.48	7	1.34	53.12	11	30.19
Bacteroidaceae		0.00	4.39	12	1.81	0.04	0	0.05
Burkholderiaceae		0.07	0.39	5	0.25	0.04	0	0.09
Coriobacteriaceae		0.00	0.20	1	0.19	7.39	9	4.30
Deferribacteraceae	0.01		2.98	12	1.43	23.94	11	24.12
Desulfo-vibrionaceae	0.07	0.00	4.24	12	2.03	0.25	2	0.49
Lachnospiraceae	0.00		13.10	12	5.98	2.32	8	3.38
Muribaculaceae	0.00	0.04	32.42	12	4.71	2.29	6	3.59
Prevotellaceae		0.00	1.00	9	0.52	0.01	0	0.02
Rikenellaceae	0.05	0.00	15.50	12	6.51	0.27	2	0.47
Ruminococcaceae	0.00		8.49	12	3.18	2.11	9	1.74
Tannerellaceae		0.00	10.63	12	8.47	0.03	0	0.05
unknown *Gastranaero-philales*		0.19	0.66	5	0.88	0.06	0	0.13

**Family level (PD)**								
Family	*p* value (W)	*p* value (F)	untreated			streptomycin treated		
			mean [%]	n	SD [%]	mean [%]	n	SD [%]
Anaero-plasmataceae		0.02	2.57	9	2.61	4.75	1	12.13
Atopobiaceae		0.00	0.26	1	0.17	54.27	8	35.40
Bacteroidaceae		0.01	4.25	11	2.26	5.89	3	14.57

Burkholderiaceae		0.00	1.17	11	0.59	0.32	1	0.58
Coriobacteriaceae		0.00	0.03	0	0.03	7.68	7	5.03
Desulfo-vibrionaceae		0.03	0.71	6	0.63	0.01	0	0.02
Erysipelotrichaceae		0.09	2.06	10	1.07	1.12	3	1.76
Lachnospiraceae	0.00		25.61	11	12.51	2.34	6	3.90
Marinifilaceae		0.00	1.95	11	0.63	1.37	2	3.20
Muribaculaceae	0.01	0.04	43.01	11	11.18	4.18	4	7.61
Prevotellaceae		0.00	1.62	9	0.69	0.04	0	0.06
Rikenellaceae	0.05	0.00	9.92	11	1.76	0.88	2	1.65
