# Supplementary material for: A diet-specific microbiota drives Salmonella Typhimurium to adapt its in vivo response to plant-derived substrates
Source: Anim Microbiome. 2021 Mar 17;3:24. doi: 10.1186/s42523-021-00082-8 (PMC7972205; doi:10.1186/s42523-021-00082-8)
Supplement: Supplementary file 6 — Additional file 6 Table S5. Significant influences of the S. Typhimurium infection on the microbiota composition. [file 42523_2021_82_MOESM6_ESM.pdf]

Table S5. Significant influences of the *S.* Typhimurium infection on the microbiota composition. W, Wilcoxon Rank Sum Test; F, Fisher's Exact Test; n, number of mice harboring species; SD, standard deviation; WD, Westernized diet; PD, plant-based diet.

**Family level (WD)**								
Family	*p* value (W)	*p* value (F)	streptomycin treated			infected		
			mean [%]	n	SD [%]	mean [%]	n	SD [%]
Coriobacteriaceae	0.00		7.39	9	4.30	4.41	10	2.49
Deferribacteraceae		0.02	23.94	11	24.12	1.79	5	2.57
Enterobacteriaceae		0.00	0.43	1	1.13	58.05	11	19.47
Lachnospiraceae		0.00	2.32	8	3.38	0.12	0	0.06
Ruminococcaceae		0.00	2.11	9	1.74	0.23	1	0.19

**Family level (PD)**								
Family	p value (W)	p value (F)	streptomycin treated			infected		
			mean [%]	n	SD [%]	mean [%]	n	SD [%]
Coriobacteriaceae	0.13		7.68	7	5.03	2.23	8	2.45
Enterobacteriaceae		0.01	9.96	2	20.75	47.51	10	28.55
Lachnospiraceae		0.01	2.34	6	3.90	0.89	1	2.52
Ruminococcaceae		0.01	2.66	6	2.15	0.69	1	2.01
