# Supplementary material for: A diet-specific microbiota drives Salmonella Typhimurium to adapt its in vivo response to plant-derived substrates
Source: Anim Microbiome. 2021 Mar 17;3:24. doi: 10.1186/s42523-021-00082-8 (PMC7972205; doi:10.1186/s42523-021-00082-8)
Supplement: Supplementary file 7 — Additional file 7 Table S6. Oligonucleotides used in this study. [file 42523_2021_82_MOESM7_ESM.pdf]

Table S6. Oligonucleotides used in this study.

name	sequence 5'-3'	gene/target	comment
Fwd invA_del	GTCGTACTATTGAAAAGCTGTCTTAATTTAATATTAACAGGATACCTATAGTGTAGGCTGGAGCTGCTTC	*invA*	deletion of *invA*
Rev invA_del	TAATTCAGCGATATCCAAATGTTGCATAGATCTTTTCCTTAATTAAGCCCCATATGAATATCCTCCTTA	*invA*	deletion of *invA*
STM3254_Del_fwd	GCGGCTGTGAAGGGCAACTGTGAGGAATCGCCTGTGATCTACACGCTAACGTGTAGGCTGGAGCTGCTTC	STM3254	deletion of STM3254
STM3254_Del_rev	TATTGTAATTTAACCTACAGCTTTCTGACGTGGATTTGCGAAAGTAAGGTCATATGAATATCCTCCTTA	STM3254	deletion of STM3254
gatR_Del-DR_fwd	GGAAGTCATCTGGCGGAAGGCGACAATCAGGAAGTCATTCTGGAAGATCGGTGTAGGCTGGAGCTGCTTC	*gatR*-HTH	deletion of *gatR*-HTH
gatR_Del_HTH_rev	GCAGCCGCCTGGGCAATCCGCTTTTTCGGATCGCTGGCGAGTTGATACCGCATATGAATATCCTCCTTA	*gatR*-HTH	deletion of *gatR*-HTH
